# Integrating Transcriptome and Chemical Analyses to Provide Insights into Biosynthesis of Terpenoids and Flavonoids in the Medicinal Industrial Crop *Andrographis paniculate* and Its Antiviral Medicinal Parts

**DOI:** 10.3390/molecules29040852

**Published:** 2024-02-14

**Authors:** Kuo Yu, Pengjie Liang, Heshui Yu, Hui Liu, Jialiang Guo, Xiaohui Yan, Zheng Li, Guoqiang Li, Ying Wang, Chunhua Wang

**Affiliations:** 1 School of Medicine, Foshan University, Foshan 528225, China; yk13752738531@163.com (K.Y.); jiellh@163.com (P.L.); tianmahui2011@163.com (H.L.); janalguo@126.com (J.G.); liguoqiang@fosu.edu.cn (G.L.); 2 College of Pharmaceutical Engineering of Traditional Chinese Medicine, Tianjin University of Traditional Chinese Medicine, Tianjin 301617, China; hs_yu08@163.com (H.Y.); yanxh@tjutcm.edu.cn (X.Y.); lizheng@tjutcm.edu.cn (Z.L.); 3 Institute of Traditional Chinese Medicine & Natural Products, College of Pharmacy, Jinan University, Guangzhou 510632, China

**Keywords:** *Andrographis paniculata*, active ingredients, chemical analysis, transcriptome analysis, molecular docking

## Abstract

*Andrographis paniculata* is a medicinal plant traditionally used to produce diterpene lactones and flavonoids, which possess various biological activities. Widely distributed in China, India, and other Southeast Asia countries, *A. paniculata* has become an important economic crop, significantly treating SARS-CoV-2, and is being cultivated on a large scale in southern China. The biosynthesis of active ingredients in *A. paniculata* are regulated and controlled by genes, but their specific roles are still not fully understood. To further explore the growth regulation factors and utilization of its medicinal parts of this industrial crop, chemical and transcriptome analyses were conducted on the roots, stems, and leaves of *A. paniculata* to identify the biosynthesis pathways and related candidate genes of the active ingredients. The chemical analysis revealed that the main components of *A. paniculata* were diterpene lactones and flavonoids, which displayed potential ability to treat SARS-CoV-2 through molecular docking. Moreover, the transcriptome sequencing annotated a total of 40,850 unigenes, including 7962 differentially expressed genes. Among these, 120 genes were involved in diterpene lactone biosynthesis and 60 genes were involved in flavonoid biosynthesis. The expression of diterpene lactone-related genes was the highest in leaves and the lowest in roots, consistent with our content determination results. It is speculated that these highly expressed genes in leaves may be involved in the biosynthesis pathway of diterpenes. Furthermore, two class Ⅰ terpene synthases in *A. paniculata* transcriptome were also annotated, providing reference for the downstream pathway of the diterpene lactone biosynthesis. With their excellent market value, our experiments will promote the study of the biosynthetic genes for active ingredients in *A. paniculata* and provide insights for subsequent in vitro biosynthesis.

## 1. Introduction

*Andrographis paniculata* is an annual herbaceous plant in the Acanthaceae family that is widely found in the tropics and subtropics, especially in China, India, Malaysia, and other places [1,2]. In China, *A. paniculata* is considered one of the “Great Southern Medicine” and is known as “natural antibiotics” for its effectiveness in treating inflammation, influenza, cancer, and other conditions [3,4]. The chemical constituents of *A. paniculata* primarily include diterpene lactones and flavonoids, with the main active ingredients being andrographolide, neoandrographolide, and deoxyandrographolide [5,6]. *A. Paniculata* has many applications in medicine and the chemical industry [7,8]. Notably, andrographolide from *A. paniculata* has been approved as a key component in many Chinese patent medicines, including andrographolide dropping pills, andrographolide tablets, and andrographolide soft capsules [9]. The highly contagious SARS-CoV-2 pandemic has caused thousands of deaths due to the unavailability of specific medications [10]. The Xiyanping injection, made from andrographolide derivatives, has been recommended for severe patients [11]. Owing to the increasing commercial value of *A. paniculata*, large-scale cultivation has been established in southern China with many good agricultural practice (GAP) planting bases [12]. However, supply remains insufficient to meet market demand. Therefore, modern biotechnology to enable in vitro biosynthesis is imperative.

Diterpene lactones are characteristic compounds in *A. paniculata*, and their upstream pathway of biosynthesis is relatively well understood, including two pathways: the mevalonic acid (MVA) pathway and the plastidial 2-C-methylerythritol 4-phosphate (MEP) pathway [13,14]. The MVA pathway produces isopentenyl pyrophosphate (IPP) and dimethylallyl pyrophosphate (DMAPP) from acetyl-CoA, while the MEP pathway synthesizes IPP and DMAPP from pyruvate and glyceraldehyde-3-phosphate. These compounds are then converted into *ent*-copalyl diphosphate (*ent*-cpp) by geranylgeranyl diphosphate synthase (GGPS) and *ent*-copalyl diphosphate synthase (*ent*-cps), with subsequent enzymes believed to be cytochrome P450 and class Ⅰ terpene synthase [15,16,17]. Although transcriptomic and genomic studies in recent years have examined the downstream biosynthesis pathway of *A. paniculata*, the key class Ⅰ terpene synthase enzymes have not been identified, leaving the pathway unclear [16,18]. Additionally, despite the abundance of flavonoids in *A. paniculate*, their biosynthesis has received scarce research attention, with limited literature on the topic available.

In order to extract the key enzymes of biosynthesis of the active ingredients in *A. paniculata* and clarify their biosynthetic pathways, a comprehensive and systematic profiling of *A. paniculata* was conducted. A qualitative analysis of different parts of *A. paniculata* was performed using HPLC-MS and found that the active ingredients are mainly diterpene lactones and flavonoids. The molecular docking of these active ingredients showed potential for treating SARS-CoV-2 and significant commercial value. Moreover, the quantitative analysis on the chemical constituents in different tissues was also performed to determine the tissue-specific differences. Concurrently, transcriptome sequencing was used to analyze *A. paniculata*. Combining DEG analysis with a quantitative analysis of the active ingredients in different tissues made it possible to speculate on the biosynthetic pathways and enzymes involved in producing active components in *A. paniculata.* This study fills a gap of flavonoid biosynthesis in *A. paniculata*, and the transcriptome analysis data will facilitate further research on the biosynthesis genes of natural products in *A. paniculata*, enriching its genetic information and providing insights for the subsequent in vitro biosynthesis of these diterpene lactones and flavonoids.

## 2. Results

### 2.1. UPLC-MS

The extracts of the leaves, stems, and roots of *A. paniculata* were scanned in the positive mode using UPLC-MS (Table 1). The results reveal that the primary chemical constituents were diterpene lactones and flavonoids. The identified diterpene lactones included andrographolide, 14-deoxyandrographolide, dehydroandrographolide, 14-deoxyandrographiside, paniculide B, 12*S*-hydroxyandrographolide, andrographolactone, bisandrographolide A, and andrograpanin. The detected flavonoids consisted of 5, 4’-dihydroxy-7, 2’, 6’-trimethoxy flavone, andrographidin A, 5-hydroxy-7, dihydroscullcapflavone I, 8-dimethoxy flavanone, apigenin-7, 5-hydroxy-7, 4’-dimethylether, and 8-dimethoxy flavone.

### 2.2. Molecular Docking

Previous studies have reported the presence of potential chemical components for treating SARS-CoV-2 in *A. paniculata* [19,20]. Therefore, 30 *A. paniculata* compounds and four proteins (*S* protein, PLpro, RdRp, and ACE2) were selected related to SARS-CoV-2 for molecular docking. The SARS-CoV-2 spike protein can bind to the human ACE2 receptor, enabling the fusion of the viral and host cell membranes, resulting in infection [21]. The PLpro and RdRP proteins are critical for viral infection and replication, making them important antiviral drug targets [22,23]. Remdesivir is an antiviral drug that treats the Ebola and Marburg viruses [24]. It has been reported as a potential drug to treat SARS-CoV-2 in recent clinical trials [25] and was thus utilized as a positive control in this experiment.

**Figure 1:**
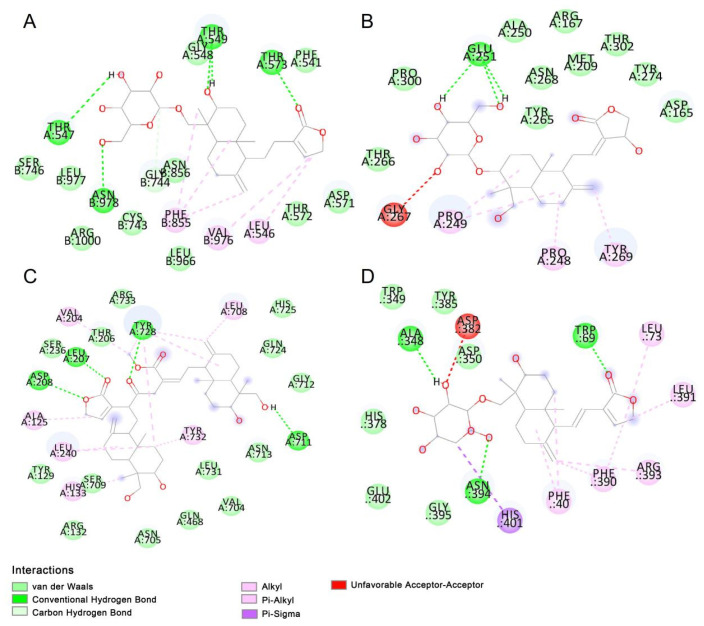
Schematic diagram of the binding between main components in *A. paniculate* and proteins by molecular docking. (**A**) 14-deoxyandrographisidedistribution docking with *S* protein. (**B**) 3-*O*-*β*-D-Glucopyranosylandrographolide docking with PLpro. (**C**) Bisandrographolide D docking with RdRp. (**D**) 14-deoxy-11-12-didehydroandrographiside docking with ACE2.

In molecular docking, binding energies below −6 kcal/mol indicate strong ligand–protein binding affinity [26]. In our research, remdesivir showed a favorable binding affinity with the spike protein (−8.3 kcal/mol), PLpro (−6.8 kcal/mol), RdRp protein (−8.0 kcal/mol), and ACE2 (−7.8 kcal/mol), suggesting that it plays a role in inhibiting SARS-CoV-2. All compounds selected from *A. paniculata* showed good docking scores of less than −6 kcal/mol with *S* protein, RdRp, and ACE2 (Table 2). However, when docking with PLpro, three compounds (andrographolactone, 14-deoxyandrographolide, and bisandrographolide A) did not meet the requirements of less than −6 kcal/mol. There were 14, 14, 8, and 11 compounds that showed better binding affinity than remdesivir when docking with *S* protein, PLpro, RdRp, and ACE2, respectively. Among these compounds, 14-deoxyandrographiside showed good binding affinity with RdRp (−6.8 kcal/mol), PLpro (−8.3 kcal/mol), and ACE2 (−7.8 kcal/mol); 14-deoxy-11-12-didehydroandrographiside showed good binding affinity with *S* protein (−8.3 kcal/mol), RdRp (−6.8 kcal/mol), PLpro (−8.3 kcal/mol), and ACE2 (−7.8 kcal/mol); 3-*O*-*β*-D-Glucopyranosylandrographolide showed good binding affinity with *S* protein (−9.0 kcal/mol), RdRp (−7.2 kcal/mol), PLpro (8.0 kcal/mol), and ACE2 (−8.0 kcal/mol). These compounds were speculated to have potential for treating SARS-CoV-2. Ligands and receptors bind through various bond energies, especially hydrogen bonds. The 2D visualization of compounds showed the various forces between protein receptors and ligands (Figure 1). In conclusion, numerous compounds in *A. paniculata* may play important roles in treating SARS-CoV-2 by preventing viral replication and transcription and by inhibiting the virus’s interaction with the human ACE2 receptor.

### 2.3. Transcriptome Sequencing and De Novo Assembly of A. paniculata

**Figure 2:**
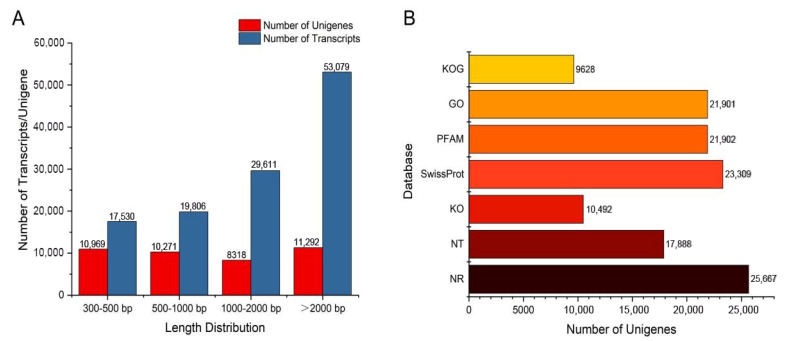
Transcription splicing and database annotation. (**A**) Transcript and unigene length distribution. (**B**) Number of gene annotations in the database.

Three different tissues (leaf, stem, and root) of *A. paniculata* were chosen with three replicates for transcriptome sequencing using the Illumina Hiseq 6000 sequencing platform. We obtained 58.2 GB of clean data, with each sample having more than 6 GB of clean data, an error rate not exceeding 0.03%, a Q20 base ratio greater than 97.63%, and a Q30 base ratio greater than 93.28% (Table S1). The sequencing quality was very high. After filtering the raw data to remove low-quality data, we used Trinity to assemble the high-quality sequences and obtained a total of 120,032 transcripts. Based on these transcripts, the sequences were further assembled, and 40,850 unigenes were obtained. The longest and shortest unigene sequences were 21,589 bp and 301 bp, respectively, with an N50 length of 1225 bp (Figure 2A; Table S2). These parameters reflect the quality of the assembled transcripts.

### 2.4. Functional Annotation of the Unigenes

We annotated 40,850 unigenes using seven databases, with the NR database having the highest number of annotations at 25667, accounting for 62.83% of all genes. This was followed by Swissprot (57.05%), NT (43.78%), KO (26.78%), Pfam (53.61%), GO (53.61%), and KOG (23.56%) (Figure 2B). In addition, 72.89% of the genes were annotated in at least one database, and 9.73% of the genes were annotated in all databases.

In the NR database, it was found that *A. paniculata* had the highest similarity with *Sesamum indicum* at 36.5%, followed by *Handroanthus impetiginosus* (17.3%), *Erythranthe guttata* (6.4%), *Quercus suber* (4.3%), *Salvia splendens* (3.4%), and others (32.1%) (Figure S1A).

The KOG database allowed us to predict and classify the functions of unigenes, with 9628 unigenes being annotated and classified into 25 groups (Figure S1B; Table S3). The largest category was Post-translational modification, protein turnover, and chaperones with 1284 unigenes, followed by General function prediction with only 1131 unigenes, and Translation, ribosomal structure, and biogenesis with 1006 unigenes.

**Figure 3:**
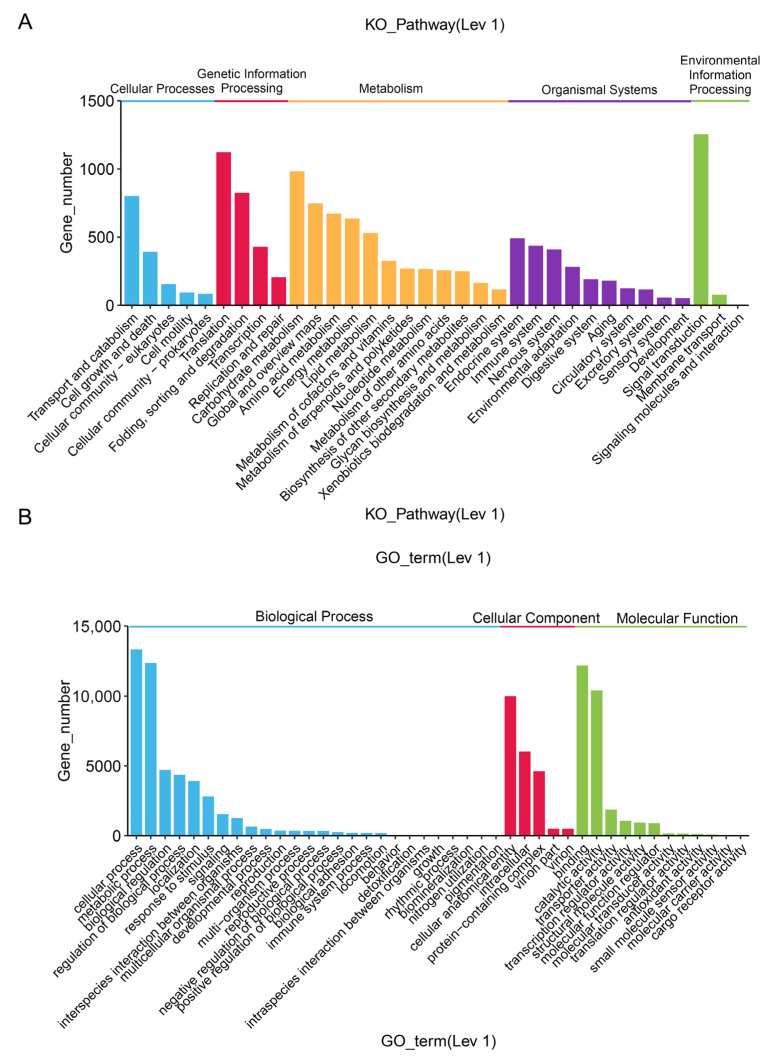
Database annotation. (**A**) KEGG classification enrichment analysis. (**B**) GO classification.

The KEGG metabolic pathways in *A. paniculata* transcriptome could be divided into four categories: environmental information processing, cellular processes, metabolism, and genetic information processing, including 24 branches and 302 metabolic pathways (Figure 3A; Table S4). The top ten pathways in annotation were Ribosome (1043), Carbon metabolism (832), Spliceosome (792), Protein processing in endoplasmic reticulum (722), Biosynthesis of amino acids (647), RNA transport (632), Endocytosis (545), Purine metabolism (467), Plant pathogen interaction (454), and Oxidative phosphorylation (447).

In the GO database, 21,901 unigenes were annotated, accounting for 53.61% of the total. According to the GO function, these were divided into three categories: molecular function, cellular component, and biological process (Figure 3B; Table S5). In the annotation results, all three types of functions were involved and were annotated in 43 second-level branches. In the molecular function category, the main branches were Binding proteins with 12,175 unigenes and Catalytic activities with 10,401 unigenes. In the cellular component category, the main branches were Cellular anatomical entity with 9997 unigenes, Intracellular with 6028 unigenes, and Protein-containing complex with 4620 unigenes. In the biological process category, the main branches were Cellular processes with 13,321 unigenes and Metabolic processes with 12,339 unigenes.

### 2.5. Differential Expression Analysis of Unigenes

**Figure 4:**
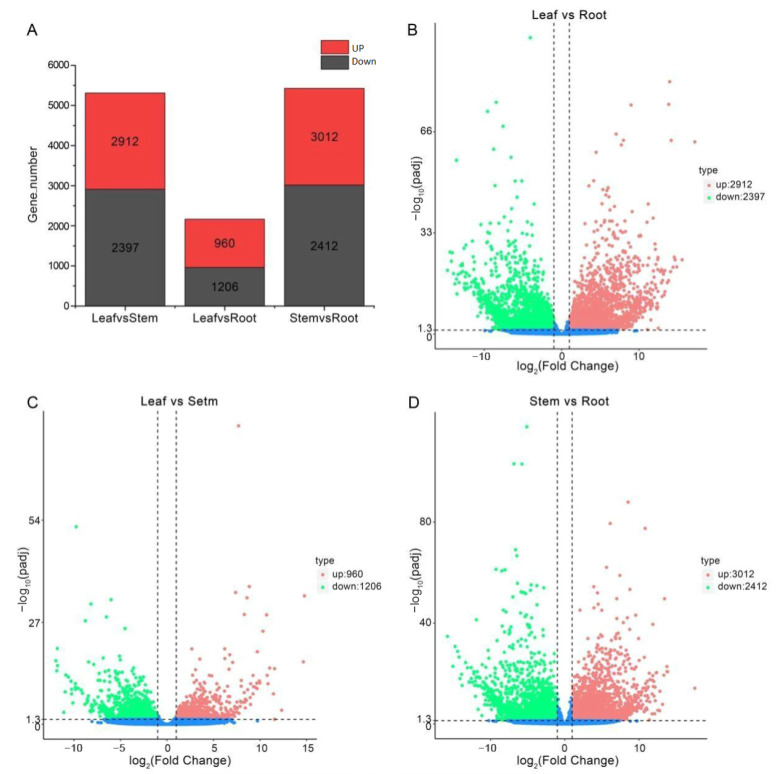
Histogram of DEGs in different tissues of *A. paniculata* and volcanic distribution map of DEGs. (**A**) Histogram of DEGs. (**B**) Volcanic map of DEGs in Leaf vs. Root. (**C**) Volcanic map of DEGs in Leaf vs. Stem. (**D**) Volcanic map of DEGs in Stem vs. Root.

To reveal transcriptional differences between tissues, we performed a differential expression analysis of the 40,850 *A. paniculata* unigenes. This identified 7962 DEGs among the leaf, stem, and root (Figure 4A). The leaf vs. root comparison had the most DEGs (5309), with 2912 upregulated in the leaf (Figure 4B; Table S6). Leaf vs. stem showed 2166 DEGs, with 960 more highly expressed in the leaf (Figure 4C; Table S7). Stem vs. root had 5424 DEGs, including 3012 upregulated in the stem (Figure 4D; Table S8). There results show little difference between the leaf and stem but more between the leaf and root and between the stem and root. This suggests that there may be a significant difference in the content of secondary metabolites between the leaf and root and between the stem and root.

**Figure 5:**
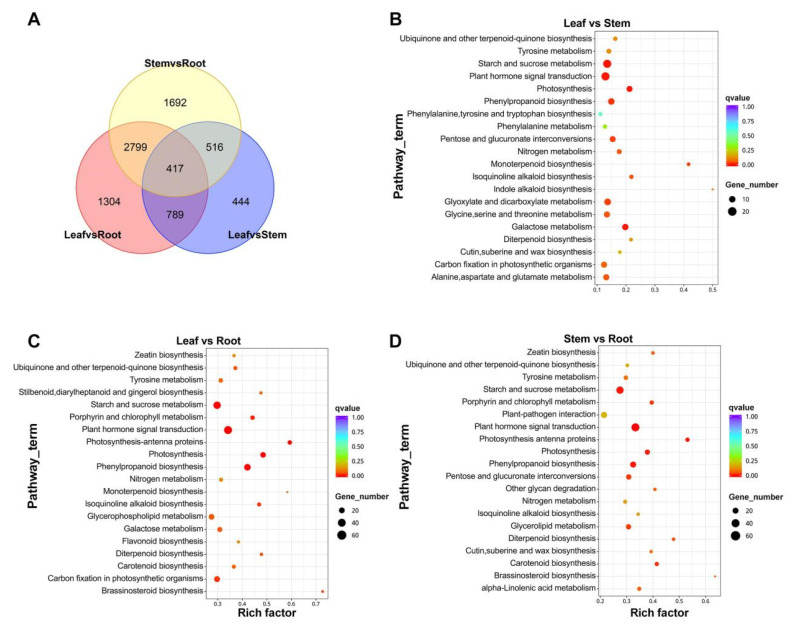
DEG analysis. (**A**) Venn diagram between the groups of *A. paniculata.* (**B**) Top 20 of KEGG pathway enrichment of DEGs in Leaf vs. Root. (**C**) Top 20 of KEGG pathway enrichment of DEGs in Leaf vs. Stem. (**D**) Top 20 of KEGG pathway enrichment of DEGs in Stem vs. Root.

There were 20,359 unigenes coexpressed in the leaf, stem, and root, while 13,173 unigenes were expressed in only one tissue (Figure S2). In the comparison of differences among groups, the leaf vs. stem, leaf vs. root, and stem vs. root groups shared 417 DEGs. (Figure 5A). In the KEGG database, 547 DEGs of the leaf vs. stem were enriched in 103 KEGG pathways (Figure 5B). The main pathways were Starch and sucrose metabolism (29), Plant hormone signal transduction (29), Glyoxylate and dicarboxylate metabolism (18), Phenylpropanoid biosynthesis (17), and Galactose metabolism (16). There were 1492 DEGs annotated in the leaf vs. root, enriched in 117 KEGG pathways (Figure 5C). The top five pathways were Plant hormone signal transduction (77), Phenylpropanoid biosynthesis (48), Photosynthesis (32), Starch and sucrose metabolism (64), and Photosynthesis-antenna proteins (19). The stem vs. root comparison had 1415 DEGs mapped to 117 KEGG pathways (Figure 5D), including Plant hormone signal transduction (75), Starch and sucrose metabolism (59), Plant–pathogen interaction (40), Phenylpropanoid biosynthesis (37), and Pentose and glucuronate interconversions (28).

### 2.6. Transcriptome Analysis of Active Ingredients

**Figure 6:**
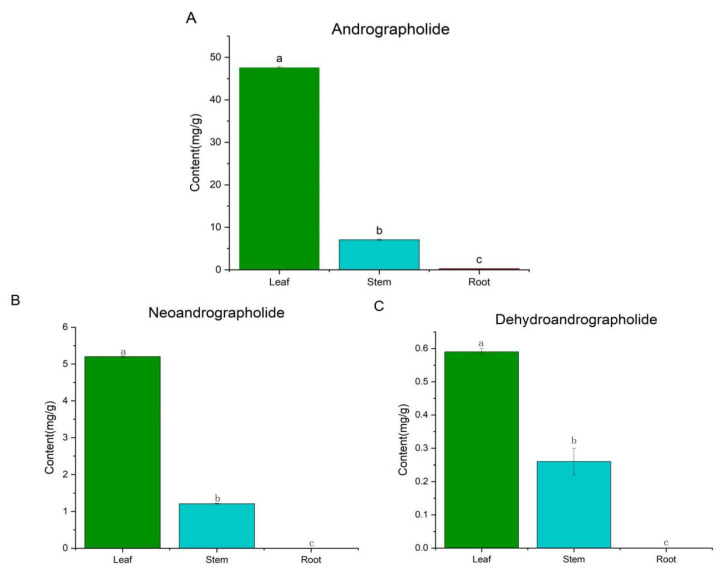
Determination of the contents of diterpene lactones. (**A**) andrographolide, (**B**) neoandrographolide, and (**C**) dehydroandrographolide. Values are expressed as means and standard deviation. Significant differences (*p* < 0.05) were analyzed using Origin2019 and are indicated by letters a, b, and c in the leaf, stem, and root.

To relate gene expression to metabolite accumulation, we quantified the major bioactive diterpenoids in *A. paniculata* tissues by using HPLC. The andrographolide content was highest in leaves (47.60 ± 0.23 mg/g), followed by stems (7.06 ± 0.17 mg/g) and roots (0.25 ± 0.01 mg/g) (Figure 6A). The diterpenoid neoandrographolide (Figure 6B) and dehydroandrographolide (Figure 6C) were also most abundant in leaves (5.20 ± 0.02 and 1.21 ± 0.01 mg/g, respectively) compared to stems (0.59 ± 0.01 and 0.26 ± 0.04 mg/g), and they were not detected in roots. These results indicate a large number of terpenoid components in the leaves and a small number in the roots of *A. paniculata.*

Terpenoid backbone and Diterpene biosynthesis are the main KEGG pathways involved in diterpene lactone biosynthesis. In contrast, the KEGG pathways involved in flavonoid biosynthesis include Flavone and flavonol biosynthesis, Flavonoid biosynthesis, Phenylalanine metabolism, and Phenylpropanoid biosynthesis. The genes annotated in these KEGG pathways are presented in Table 3.

#### 2.6.1. Diterpene Biosynthesis

**Figure 7:**
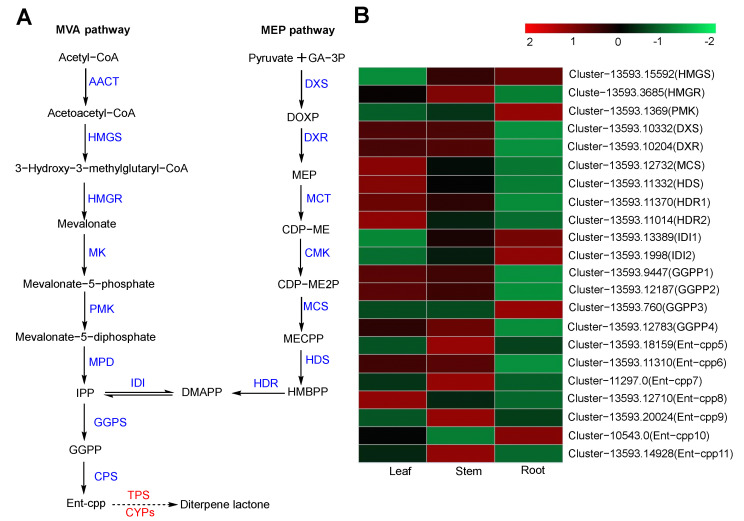
Biosynthesis pathway of diterpene lactones and the heatmap analysis of their DEGs. (**A**) Predicting the biosynthetic pathway of diterpene lactones. Solid arrows are known steps, and unknown ones are indicated by dashed arrows. Enzyme names are known with blue and unknown with red. (**B**) Heatmap of diterpene lactone pathway DEGs.

Diterpene lactones such as andrographolide, neoandrographolide, and dehydroandrographolide are characteristic compounds in *A. paniculata*. Current research shows that DMAPP and IPP, which are precursors of terpene biosynthesis, are biosynthesized by the MVA and MEP pathways (Figure 7A) [13,14]. The MVA pathway uses acetyl CoA as the raw material. It generates DMAPP and IPP through the action of several enzymes, including acetoacety-CoA thiolase (AACT), 3-hydroxy-3-methylglutary-1 CoA synthase (HMGS), 3-hydroxy-3-methylglutary CoA reductase (HMGR), mevalonate kinase (MK), phosphomevalonate kinase (PMK), mevalonate pyrophosphate (MVD), and ispentenyl diphosphate isomerase (IDI). The MEP pathway utilizes pyruvate and G3P as raw materials and involves enzymes such as 1-deoxy-D-xylulose, 5-phosphate synthase (DXS), 1-deoxy-D-xyluloses-phosphatereduetoisomerase (DXR), 2-c-methyl-d-erythrol 4-phosphatecytidyltransferase (MCT), 4-diphosphocytidyl-2-C-methyl-D-erythritol kinase (CMK), 2-C-methyl-D-erythritol-2,4-cyclodiphosohate synthase (MCS), 1-hydroxy-2-methyl-2-(E)-butenyl-4-diphosphate synthase (HDS), and isopentenyl monophosphate kinase (IPK). Research has demonstrated that diterpene lactone biosynthesis primarily proceeds via the MEP pathway [27]. The precursors DMAPP and IPP condense in a 1:3 ratio by geranylgeranyl diphosphate synthase (GGPS) to form geranylgeranyl diphosphate (GGPP) [28]. GGPP is subsequently converted to *ent*-copalyl diphosphate (*ent*-CPP) by *ent*-copalyl diphosphate synthase (*ent*-CPS) [29]. However, the downstream enzymatic steps remain unclear. Based on the biosynthetic pathways elucidated in other diterpene-producing plants, such as *paclitaxel* [30], *Tripterygium wilfordii* [31], and *Salvia miltiorrhiza* [32], it is hypothesized that *ent*-CPP undergoes dephosphorylation by a kaurene synthase-like (KSL) diterpene synthase followed by successive oxidations mediated by various CYP450 enzymes to yield the diverse diterpene lactones [16,33,34].

**Figure 8:**
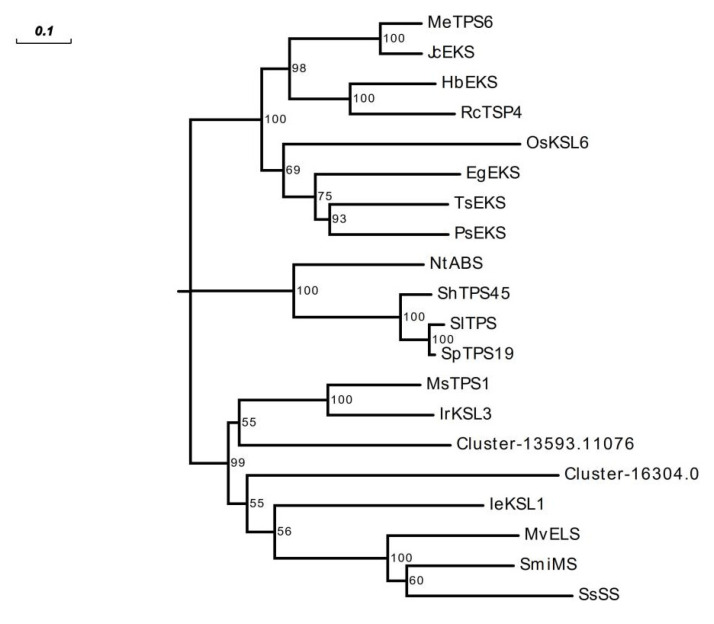
The phylogenetic relationship of class Ⅰ terpene synthase in *A. paniculata* with class Ⅰ terpene synthase in other species. The evolutionary tree was constructed by using MEGA X 10.2.1 software, and the sequence alignment was performed by using muscle comparison, and the evolutionary tree was the Neighbor-Joining method.

According to the transcriptome analysis results, 120 genes were annotated to the above diterpene biosynthetic pathways, including 22 DEGs (Figure 7B; Table S9). A heat map was drawn based on the FPKM values of DEGs in *A. paniculata*, showing differences in the expression of diterpene lactone biosynthesis-related genes in the leaf, stem, and root. The highest expression levels were found in the leaf, followed by the stem and the root. This is consistent with our content determination results, which show that diterpene lactones were most abundant in the leaf and least abundant in the root. In the MEP pathway, we annotated 6 DXS, 16 DXR, 4 MCT, 1 MCS, 1 CMK, 3 HDS, and 9 HDR genes. Among these, DXS (Cluster-13593.10332), DXR (Cluster-13593.10204), MCS (Cluster-13593.12732), HDS (Cluster-13593.11332), and HDR (Cluster-13593.11370 and Cluster-13593.11014) genes exhibited a higher expression in the leaves compared to the stems and roots, with FPKM values of 251.48, 51.52, 24.32, 286.7, 80.04, and 462.39, respectively. These enzymes may represent key nodes in diterpene lactone biosynthesis. Similarly, we annotated 13 *GPPS* and 33 *ent*-CPS in the downstream pathway. Among these, *Ent*-CPS (Cluster-13593.11310 and Cluster-13593.12710) and GGPS (Cluster-13593.12187 and Cluster-13593.9447) were highly expressed in the leaves and are speculated to be involved in the downstream biosynthesis of diterpene lactones. *Ent*-CPP requires dephosphorylation by a class Ⅰ terpene synthase. Mining of the *A. paniculata* transcriptome revealed two class I terpene synthases (Cluster-16304.0 and Cluster-13593.11076) containing the characteristic DDXXD motif (Figure S3). Both terpene synthase genes displayed a high leaf expression but a deficient expression in the roots. A phylogenetic analysis of these two genes and terpene synthases from other species (Table S10) is shown in Figure 8. The diterpene skeletons were further modified into diterpenes primarily through CYP450. Thus, diterpene lactones in *A. paniculata* are considered to be dependent on CYP450 [35]. We annotated 258 CYP450 in the transcriptome, consistent with other angiosperms (Table S11) [36]. The CYP71 and CYP76 families have been demonstrated to participate in labdane-related diterpenoid biosynthesis in other species [37]. A total of 64 CYP71 and 15 CYP76 genes were annotated from the *A. paniculata* transcriptome, exhibiting high expression in leaves.

#### 2.6.2. Flavonoid Biosynthesis

**Figure 9:**
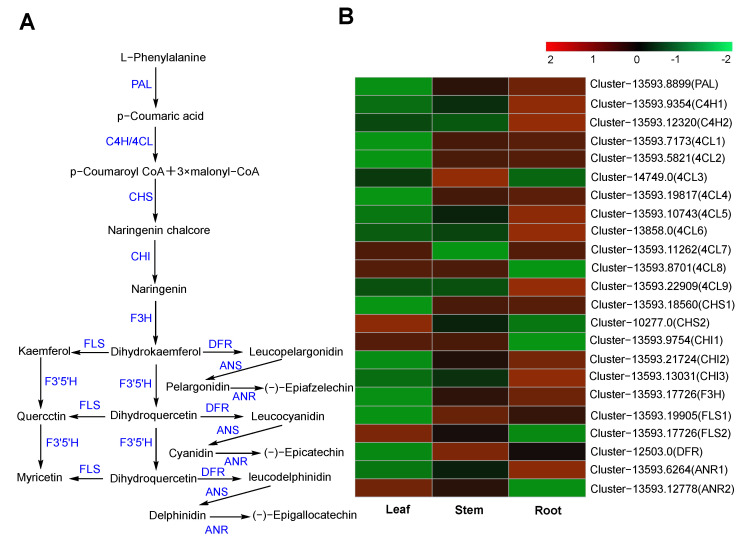
Biosynthesis pathway of flavonoid biosynthesis and the heatmap analysis of their DEGs. (**A**) Predicting the biosynthetic pathway of flavonoids. The steps and enzymes shown in the diagram are known. (**B**) Heatmap of flavonoid pathway of DEGs.

Flavonoids are abundant compounds in medicinal plants, and their biosynthetic pathway is relatively well characterized. The flavonoid metabolic pathway utilizes L-phenylalanine as a substrate and generates dihydrokaemferol through the action of phenylalanine ammonia lyase (PAL), *trans* cinnate 4-hydroxylase (C4H), 4-coumarate CoA ligase (4CL), chalcone synthase (CHS), chalcone isomerase (CHI), and flavanone-3-hydroxylase/naringenin 3-dioxygenase (F3H). Dihydrokaemferol is then modified by enzymes such as flavonol synthase (FLS), flavanoid 3′,5′-hydroxylase (F3′5′H), bifunctional dihydroflavonol 4-reductase/flavanone 4-reductase (DFR), anthocyanidin synthase (ANS), and anthocyanidin reductase (ANR) to yield myricetin, (-)-Epigallocatechin, and other flavonoids [38,39] (Figure 9A).

Although numerous flavonoids have been isolated from *A. paniculata*, flavonoid biosynthesis in this species remains unreported. Here, we mined the *A. paniculata* transcriptome and identified 60 candidate genes involved in the flavonoid pathway, including 23 DEGs (Figure 9B; Table S12). Most genes were highly expressed in the root, and least expressed in the leaf, which is consistent with previous research showing that the content of flavonoids is high in roots [40]. Among these genes, PAL (Cluster-13593.8899), C4H (Cluster-13593.9354 and Cluster-13593.9354), 4CL (Cluster-13593.7173, Cluster-13593.5821, Cluster-13593.19817, Cluster-13593.10743, Cluster-13858.0, and Cluster-13593.22909), CHS (Cluster-13593.18560), CHI (Cluster-13593.21724 and Cluster-13593.13031), F3H (Cluster-13593.17726), and ANR (Cluster-13593.6264) were significantly upregulated in the root, suggesting that these enzymes may be critical in the biosynthesis of flavonoids.

### 2.7. Identification of SSRs

**Figure 10:**
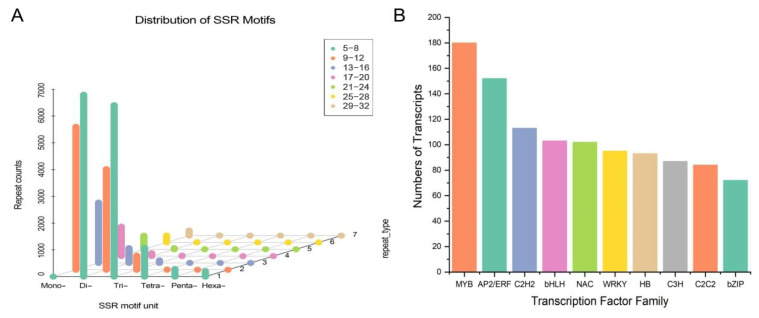
SSR and TF analyses of transcripts. (**A**) Quantitative distribution of different motif lengths and repeats in SSR of *A. paniculata.* (**B**) Top 10 of TF family distribution histogram.

SSRs, or microsatellites, are widely distributed in eukaryotic genomes [41]. We analyzed the 40,850 unigenes in *A. paniculata* transcriptome using MISA 1.0 software and identified a total of 29,751 SSRs. More than 1 SSR was found in 7264 unigenes, and compound SSRs were found in 4129 unigenes (Figure 10A). There were 9817 mono-nucleotide repeats, 11,294 di-nucleotide repeats, 7051 tri-nucleotide repeats, and 1589 hexa-, penta-, and tetra-nucleotide repeats. The number of SSR units in the *A. paniculata* transcriptome were distributed between 5 and 32 times. There were 27,333 SSR units with repeat lengths between 5 and 16 times, accounting for 91.87% of the total repeat units. There were 2241 SSRs with repeat lengths between 17 and 32 times, accounting for 7.53% of the total SSRs. There were also 177 SSRs with repeat lengths greater than 32, with mono-nucleotide repeats being the most common. This SSR landscape provides a rich basis for developing microsatellite markers to study genetic diversity, population structure, and linkage mapping in *A. paniculata*.

### 2.8. Transcription Factor Analysis

Transcription factors (TFs) can activate or inhibit functional gene expression in the biosynthesis pathway, thereby regulating the synthesis and accumulation of plant secondary metabolites [42,43]. We used iTAK 1.2 software to analyze the TFs of *A. paniculata*. The results show that a total of 2180 unigenes were annotated as TFs (Figure 10B), belonging to 73 transcription factor types. The most abundant families were 180 MYB (v-myb avian myeloblastosis virtual oncogene homolog), 152 AP2/ERF-ERF (APETAL2/ethylene-responsive factor), and 113 C2H2 (Cys2-His2). The large number of MYB TFs in *A. paniculata* suggests complex regulatory control over secondary metabolism.

### 2.9. qRT-PCR Analysis

**Figure 11:**
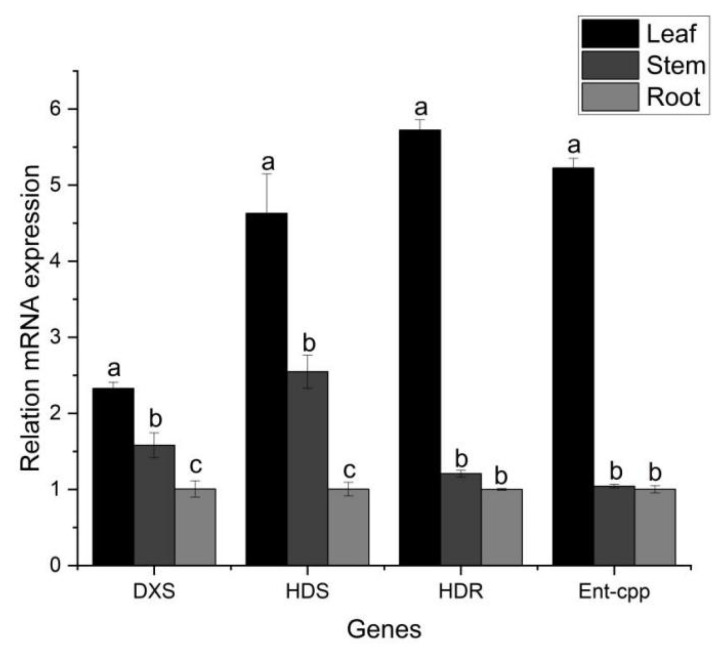
Quantitative PCR detection of genes for terpenoid skeleton synthesis in *A. paniculata.* Values are expressed as means and standard deviation. Significant differences (*p* < 0.05) were analyzed using Origin2019 and are indicated by letters a, b, and c in the leaf, stem, and root.

To validate the transcriptome results, we performed qRT-PCR of four key DEGs from terpenoid backbone biosynthesis (DXS, HDS, HDR, and *Ent*-cps) in leaves, stems, and roots (Figure 11). The qRT-PCR expression profiles matched the FPKM abundance patterns from the transcriptome data. This verification by an independent method provides confidence in the transcriptome analysis pipeline utilized in this study. Moving forward, the transcriptional landscape revealed by our integrative transcriptome and chemical profiling will guide further investigations into regulating medicinally important terpenoids and flavonoids in *A. paniculata*.

## 3. Discussion

*A. paniculata* is an industrial crop with significant potential in the medical and pharmaceutical industries [7,8]. It has been reported that *A. paniculata* effectively inhibited the spread of Indian influenza in 1919 [6] and has demonstrated therapeutic effects on SARS-CoV-2 [44]. As of 6 June 2022, SARS-CoV-2 had affected over 530 million people worldwide and resulted in more than 1 million deaths [45]. Hence, it is urgent to find specific drugs for the treatment of SARS-CoV-2. This study performed molecular docking studies on the active ingredients in *A. paniculata* and SARS-CoV-2-related proteins, including *S* protein, RdRp, ACE2, and PLpro. Most of the compounds in *A. paniculata* showed low binding energy with the *S* protein and PLpro, suggesting that the active ingredients in *A. paniculata* may inhibit the combination of the virus and the human body by binding with these two proteins [46]. The results of our research suggest that the active compounds in *A. paniculata* could be used as industrial raw materials to produce potential drugs for treating SARS-CoV-2, which has significant economic value. At present, *A. paniculata*-related drugs are mainly made from its crude extract or purified andrographolide and other ingredients; however, due to some of the protein and other components that may remain in it, it may cause mild toxic side effects such as allergic reactions, skin diseases, and gastrointestinal diseases. Therefore, the study of the biosynthesis pathway of *A. paniculata’s* active ingredients is conducive to the subsequent production of related drugs [47,48,49] under the premise of clarifying the main active ingredients, using gene-editing technology to knock out some side reactions, thereby reducing the generation of by-products. Furthermore, through synthetic biology technology, we can construct genes for the main biosynthetic pathways and use chassis cells to synthesize the target compound, thereby reducing and eliminating the production of side components.

The present study utilized UPLC-MS to analyze the leaf, stem, and root of *A. paniculata.* The results reveal that the major bioactive constituents were diterpene lactones and flavonoids, including andrographolide, dehydroandrographolide, and neoandrographolide, which have been reported to exhibit antiviral, anti-inflammatory, and immunomodulatory properties [50,51]. A quantitative HPLC analysis further quantified the tissue-specific accumulation patterns of these terpenoids, indicating the highest abundance in leaves and the lowest levels in roots. Given that the aboveground parts of *A. paniculata* are commonly utilized in Chinese herbal medicine, our findings suggest that the leaves may serve as an optimal industrial source for efficient extraction and an enhanced yield of terpenoids. While standards of several *A. paniculata* flavonoids were obtained and targeted for HPLC analysis, none were detected. This contrasts with recent evidence demonstrating substantial flavonoid accumulation in roots [40]. Our study also lacks the ability to detect the accumulation of relevant active flavonoids in different tissues to complement and elucidate their metabolic pathways. Further isolation and characterization of flavonoids from the examined variety will help elucidate their contribution to the phytochemical landscape of *A. paniculata* antiviral tissues. Overall, the integrated metabolomic and transcriptomic analyses provide valuable insights into the tissue-specific biosynthesis and medicinal composition of terpenoids and flavonoids in *A. paniculata*. The knowledge of biosynthetic genes and accumulation patterns for bioactive phytochemicals will inform future metabolic engineering efforts to enhance the production of antiviral compounds.

Transcriptome sequencing technology has become an indispensable approach for comprehensively analyzing non-model medicinal plants like *A. paniculata* [52]. In the absence of a complete reference genome [16,53], we utilized a de novo assembly with Trinity to generate the *A. paniculata* leaf, stem, and root transcriptomes. This yielded 120,032 transcripts and 40,850 unigenes, providing a valuable genomic resource. Upon the availability of the complete *A. paniculata* genome, the transcript assemblies could be further improved. Through transcriptome annotation, we identified 120 and 60 genes that were putatively involved in diterpene lactone and flavonoid biosynthesis, respectively. The predominant expression of diterpene lactone genes in leaves concurred with our metabolite profiling showing abundant lactone accumulation in leaves. These transcriptomic patterns suggest the involvement of these genes in tissue-specific terpenoid and flavonoid production. Diterpene lactone biosynthesis in *A. paniculata* proceeds via cyclization and rearrangement reactions catalyzed by class II and class I terpene synthases [34]. While class II terpene synthases have been functionally characterized in vitro and in vivo [15], class I terpene synthases remain to be fully elucidated [16]. From the *A. paniculata* transcriptomes, we mined 43 putative terpene synthase genes based on sequence conservation, translation to full-length proteins, and tissue expression patterns. Two class I terpene synthase candidates were prioritized for further functional validation to determine their role in lactone biosynthesis.

*A. paniculata* flavonoids also exhibit anti-platelet, anti-thrombotic, and anti-oxidant activities [54,55]. While the flavonoid pathway is established in other medicinal plants [38,39], the responsible genes in *A. paniculata* have not been reported. Our integrated transcriptome and metabolite datasets provide the first insights into the transcriptional regulation of *A. paniculata* flavonoid metabolism. In summary, the elucidated genes offer targets to engineer enhanced terpenoid and flavonoid production in this antiviral medicinal crop.

Integrated transcriptomic and metabolomic analyses are powerful approaches to elucidate the biosynthetic pathways of medicinal plant natural products [56]. Deciphering the underlying genetics of metabolite accumulation can provide fundamental insights into pathway regulation and resources for industrial-scale production or generation of novel bioactive compounds [57,58]. The present study identified putative genes associated with terpenoid and flavonoid metabolism in *A. paniculata*. Based on their tissue-specific expression patterns, several terpene synthase and flavonoid biosynthesis genes were revealed as candidates contributing to generating lactone and flavonoid phytochemical pools. However, further functional validation is required to conclusively determine the roles of these genes in governing terpenoid and flavonoid accumulation. Future studies should focus on enzyme assays in vitro and genetic manipulations in vivo to characterize the biosynthetic functions of the nominated genes. Elucidation of their metabolic contributions will provide the knowledge base to engineer an optimized production of antiviral phytochemicals in this important medicinal crop. Ultimately, the integrated multi-omics approaches implemented here serve as a framework to uncover biosynthetic pathways and enable biotechnological innovations in non-model plant natural product systems.

## 4. Materials and Methods

### 4.1. Plant Materials

**Figure 12:**
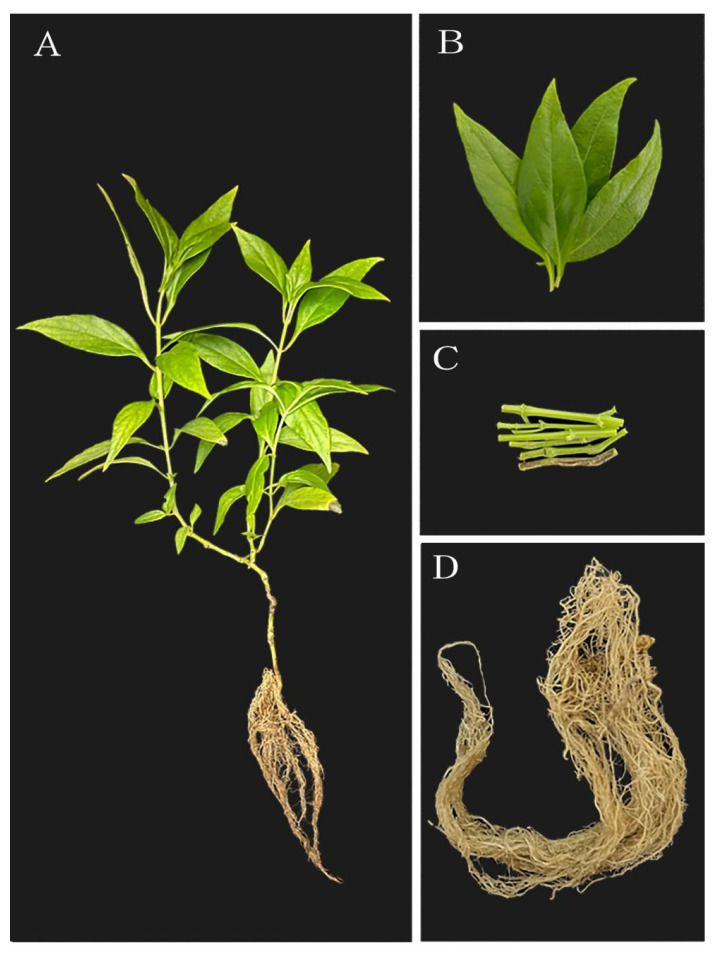
Plant and tissues of *A. paniculata*. (**A**) *A. paniculata* plant. (**B**) Leaves. (**C**) Stems. (**D**) Roots.

The plants of *A. paniculata* were collected in April 2021 from Baise city, Guangxi province, China and cultivated in the Tianjin University of Traditional Chinese Medicine. The samples were identified by Dr. Wang Chunhua and preserved in the laboratory. Fresh organs (leaf, stem, and root) were selected from three-month-old *A. paniculata* plants (Figure 12). The samples were initially rinsed with pure water, followed by sterile RNAse-free water, and the tissues were quickly dissected into small pieces (each tissue had three biological replicates, a total of nine samples) and rapidly frozen in liquid nitrogen for subsequent transcriptome experiments. For chemical analysis, fresh leaves, stems, and roots of *A. paniculata* were collected, freeze-dried, and ground into powder with mortar.

### 4.2. UPLC-MS

The powder samples were weighed to an accuracy of 0.1 g and subjected to ultrasonic extraction for 30 min (power 250 W, frequency 40 kHz) using 5 mL of methanol after a 2 h soaking period. The samples were then centrifuged at a speed of 12,000 r/min at a temperature of 4 °C for 15 min. The UPLC (Waters,, Milford, MA, USA) coupled with Q Exactive Plus-Orbitrap MS (Thermo Fisher Scientific, Waltham, MA, USA) was utilized for chromatographic detection. The chromatography column employed was Waters Acquity UPLC BEH C_18_ (100 mm × 2.1 mm, 1.7 m, Waters, USA), with a mobile phase of 0.1% formic acid (solvent A) and methanol (solvent B), 0.2 mL/min flow rate, and column temperature of 30 °C. The injection volume was 1 μL, and gradient elution was carried out as follows: 0–12 min, 5%–50% A; 12–34 min, 50%–95% A; 34–35 min, 95% A; and 35–38 min, 95%–5% A. MS parameters were ion source was ESI; positive and negative modes; 100–1500 *m*/*z* full QE scan mode; 40 arb sheath gas flow rate; 15 arb aux gas flow rate; 320 °C capillary temperature; 0.2 s scan time; 3.5 kv spray voltage; 350 °C auxiliary gas heater temperature; 70,000 full MS resolution, and 17,500 dd-MS^2^ resolution [59].

### 4.3. Molecule Docking

The 3D structures of ligands were obtained from PubChem https://pubchem.ncbi.nlm.nih.gov/ (accessed on 5 May 2022) and converted to the appropriate format with openbabel 2.4.1. These ligands were then processed using AutoDock tools 1.5.6 to add hydrogens, determine the ligand’s root, select its rotatable bonds, and store them for subsequent docking research. Similarly, the 3D structures of the spike protein (*S* protein), RdRp, ACE2, and PLpro were retrieved from PDB htpps://www.rcsb.org/ (accessed on 5 May 2022), with PDB IDs 6VSB, 6NUS, 1R42, and 4OVZ, respectively [19,60]. PyMOL 2.5.0 was used to remove water and ligands from the protein structures, and AutoDock tools 1.5.6 was used to add hydrogen, calculate charge, add atomic type, and perform other operations. The Autodock Vina 1.1.2 was employed for molecular docking, and the resulting data were analyzed using PyMOL 2.5.0 and BIOVIA Discovery Studio 2020.

### 4.4. Analysis of Different Tissue Components

Referring to the extraction method and chromatographic analysis conditions of Xia et al. [61], powder samples from different tissues (0.1 g) were extracted using ultrasonic extraction for 0.5 h (250 W power, 40 kHz frequency) with 10 mL methanol, and then these extracts were centrifuged for 15 min at 12,000 rpm and 4 °C. The supernatants were filtered through 0.22 μm membranes for HPLC analysis. Each tissue’s extraction procedure was repeated in triplicate to quantify chemical components. A Waters Arc 2998 (Waters, USA) was used to measure the content of diterpene lactones including andrographolide, neoandrographolide, and dehydroandrographolide. Standard references were obtained from Yuanye Bio-Technology Co., Ltd. (Shanghai, China), with batch numbers L23S6Y3682 (andrographolide, content ≥ 98%), Y27J8S40807 (neoandrographolide, content ≥ 98%), and R17J10F80032 (dehydroandrographolide, content ≥ 98%). The chromatography column used was Caprisil C18-P (250 × 4.6 mm, 5 μm, West Berlin, NJ, USA), with a mobile phase of water (solvent A) and acetonitrile (solvent B). The flow rate was 1 mL/min; the column temperature was 30 °C; the injection volume was 12 μL, and the detection wavelengths were 224 nm and 254 nm. Gradient elution was as follows: 0–5 min, 95%–80% A; 5–15 min, 80%–70% A; 15–25 min, 70%–66% A; 25–30 min, 66%–63% A; 30–45 min, 63%–61% A; 45–55 min 61%–5% A; 55–60 min 5%–95% A; and 60–65 min 95% A. The standard curve equation for andrographolide was Y = 27,747X − 134,819R^2^ = 1), with a linear range of 0.00265 mg/mL to 0.678 mg/mL. The standard curve equation for neoandrographolide was Y = 6985.1X − 2435.6 (R^2^ = 1), with a linear range of 0.00305 mg/mL to 0.195 mg/mL. The standard curve equation for dehydroandrographolide was Y = 16,259X − 5782.5 (R^2^ = 0.9999), with a linear range of 0.00207 mg/mL to 0.06625 mg/mL.

### 4.5. Transcriptome Sequencing and Functional Annotation

The RNA Prep Pure Plant kit (Tiangen Biotech, Beijing, China) was used to extract total RNA from the leaf, stem, and root of *A. paniculata* (three biological replicates per tissue). The concentration and integrity of RNA samples were assessed using an Aglient 2100 bioanalyzer (Agilent Technologies, Santa Clara, CA, USA). Nine RNA samples from *A. paniculata* that met the transcriptome sequencing requirements were sent to Nuohe Zhiyuan Biotechnology Co., Ltd, Shanghai, China. for sequencing on the Illumina Novaseq 6000 platform. The raw data were cleaned to remove adapters, N bases, and low-quality reads. The GC percentage, Q20, and Q30 were also calculated. Clean reads were de novo assembled into transcripts and unigenes using Trinity 2.4.0 software [62]. The blast2go tool [63] was used to perform functional annotation against various databases, including NCBI non-redundant protein sequences (NR), NCBI nucleoside sequences (NT), KEGG, GO, euKaryotic Ortholog Groups/Clusters of Orthologous Groups of proteins (KOGs), Pfam, and Swiss prot. The iTAK 1.2 software [64] identified transcription factors (TFs) in *A. paniculata*.

### 4.6. Different Expression Analysis

The gene expression levels were estimated by calculating FPKM (the number of reads per million reads from the comparison to a gene per thousand base length) values. DESeq2 [65] performed differential expression analysis for each unigene across different tissues. Different expression genes (DEGs) were identified using a screening criterion of |log2(FoldChange)| > 1 and *p* < 0.005, as determined by DESeq2. The DEGs were performed on the KEGG pathway and GO function enrichment analysis. Genes relevant to diterpene lactone and flavonoid biosynthesis were identified by combining known biosynthetic pathways with the functional annotation database results. FPKM values were used to quantify expression levels and predict related compounds’ biosynthetic pathway. Heat maps of the biosynthesis pathway and gene expression were generated using ChemDraw 20.0 and online tools https://magic.novogene.com/customer/main#/login (accessed on 11 July 2021), respectively.

### 4.7. Simple Sequence Repeat (SSR) Prediction

The MIcroSAtellite (MISA) tool http://pgrc.ipk-gatersleben.de/misa (accessed on 11 July 2021) [66] was used to detect SSRs in the unigenes of the transcriptome data. The criteria for SSR detection were a minimum of 10 repeats for mono-nucleotide, 6 for di-nucleotide, and 5 for tri-nucleotide. For four-, five-, and six-nucleotides, a minimum of 5 repeats were required. The resulting SSR data were classified and statistically analyzed.

### 4.8. Quantitative Real-Time PCR

The total RNAs were extracted from three organs (leaf, stem, and root) of *A. paniculata* using Trizol reagent. The quality of the RNA was assessed by analyzing the A260/280 ratio and resolving on 1.2% (*w*/*v*) agarose gel. The expression of the representative terpenoid genes in *A. paniculata* was detected by qRT-PCR. Four candidate gens (*DXS* Cluster-13593.10332, *HDS* Cluster-11332, *HDR* Cluster-11370, and *Ent*-cpp Cluster-13593.11310) were selected, and qRT-PCR primers were designed based on nucleotide sequences (Table S13). A QuantStudio 1 Real-Time PCR (Applied Biosystems, Carlsbad, USA) performed the qRT-PCR reactions with three replicates for each sample. The 10 μL qRT-PCR reaction mixture contained 5 μL of 2× SYBR Green I mix, 0.3 μL each forward and reverse primers, 2 μL cDNA template, and 2.4 μL ddH2O. The qRT-PCR parameters were as follows: 94 °C for 10 min (initial denaturation); 40 cycles at 95 °C for 5 s, annealing at 60 °C for 20 s, and extension at 72 °C for 10 s. The melting curve conditions were 60 °C for 8 s and 95 °C for 8 s, followed by 1 cycle. The results were analyzed using 2^−ΔΔCt^ method with *actin* (KJ494921.1) as an endogenous control [67].

## 5. Conclusions

In this study, molecular docking revealed the potential antiviral efficacy of *A. paniculata* phytochemicals against SARS-CoV-2. At present, the active effect of *A. paniculata* on SARS-CoV-2 is still focused on computational investigation, and more pharmacology and toxicology studies are needed in the future. Through transcriptome and chemical analyses, we investigated the genes that regulate the active components in different tissues of *A. paniculata*. A total of 120 and 60 genes were annotated as being putatively involved in the biosynthesis pathway of diterpene lactones containing 22 DEGs and flavonoids containing 23 DEGs, respectively. This transcriptomic dataset provides valuable insights into the transcriptional regulation governing the accumulation of antiviral terpenoids and flavonoids in different tissues of *A. paniculata*. These transcripts enrich the metabolic information base of the active substances in *A. paniculata* and provide resources for the subsequent analysis of the full-length genes of related biosynthetic enzymes. The nominated biosynthetic genes offer targets for metabolic engineering efforts to optimize the production of bioactive phytochemicals. More broadly, the multi-omics framework implemented here serves as a model for the dissection of specialized metabolism in non-model medicinal plants. These findings lay the foundation for biotechnological innovation and advanced research into the metabolism, molecular breeding, and in vitro biosynthesis of *A. paniculata* terpenoids and flavonoids. Furthermore, multi-omics techniques such as genomics, lipidomics, and metabolomics can be used to identify and validate specific functional enzymes, providing valuable active enzymes for the biosynthesis of active ingredients in *A. paniculata*.

## Figures and Tables

**Table 1:** Result of UPLC-MS in *A. paniculata.*

Group	Compounds	Retention Time	Fomula	Measured Mass (*m/z*)	MS/MS Fragment
Diterpene	12*S*-Hydroxyandrographolide	12.09	C_20_H_32_O_6_	369.1532	351.2167, 333.2059, 315.1956, 297.1852, 285.1850, 257.1555,
	Paniculide B	12.48	C_15_H_20_O_5_	281.1375	263.1280, 245.1176, 217.1226
	Dehydroandrographolide	12.73	C_20_H_28_O_4_	333.2060	333.2062, 315.1956, 297.1851, 285.1847, 257.1542
	Andrographolide	14.03	C_20_H_30_O_5_	351.2169	333.2058, 315.1975, 297.1849, 285.1849, 257.1534
	14-Deoxyandrographiside	15.49	C_26_H_40_O_9_	497.2746	335.2223, 317.2115, 299.2009, 287.2008
	Andrographolactone	16.35	C_20_H_24_O_2_	297.1852	269.1539
	14-Deoxyandrographolide	20.53	C_20_H_30_O_4_	335.2216	335.2221, 317.2110 299.2006, 287.2007 259.1695
	Bisandrographolide A	22.23	C_40_H_56_O_8_	665.4039	315.1956, 297.1852, 285.1852, 257.1539
	Andrograpanin	24.16	C_20_H_30_O_3_	319.2269	301.2164, 289.2164, 261.1848
Flavonoid	Andrographidin A	15.14	C_23_H_26_O_10_	463.1220	301.1070, 197.0445
	5, 4’-Dihydroxy-7, 8-dimethoxyflavone	17.90	C_17_H_14_O_6_	315.0864	197.0447, 119.0857
	Dihydroscullcapflavone I	21.88	C_17_H_16_O_6_	317.1020	197.0443, 121.5874
	5-Hydroxy-7, 8-dimethoxy flavanone	23.69	C_17_H_16_O_5_	301.1069	197.0443, 105.0333
	Apigenin-7,4’-dimethylether	25.69	C_17_H_14_O_5_	299.0915	167.1066, 133.1014,
	5-Hydroxy-7, 2’, 6’-trimethoxy flavone	26.92	C_18_H_16_O_6_	329.1032	167.0705, 163.1516

**Table 2:** Docking results of *A. paniculata.* The words in red are those with a binding energy greater than remdesivir.

Group	Compounds	*S* Protein Binding Affinity (kcal/mol)	PLpro Binding Affinity (kcal/mol)	RdRp Binding Affinity (kcal/mol)	ACE2 Binding Affinity (kcal/mol)
Positive control	Remdesivir	−8.3	−6.8	−8.0	−7.8
Diterpenes	Andrographolide	−8.0	−6.1	−7.7	−7.6
	Andrograpanin	−7.8	−6.4	−7.7	−7.2
	14-Deoxyandrographiside	−9.0	−6.8	−8.2	−8.2
	14-Deoxyandrographolide	−8.0	−5.9	−7.7	−7.7
	14-Deoxy-11-12-didehydroandrographiside	−8.3	−6.8	−8.3	−8.8
	Neoandrographolide	−8.9	−6.6	−7.7	−7.2
	Dehydroandrographolide	−8.1	−7.4	−7.2	−7.0
	Andropanolide	−8.0	−6.4	−7.9	−7.2
	Bisandrographolide A	−9.0	−5.8	−7.9	−8.4
	Bisandrographolide D	−9.0	−6.6	−8.9	−8.0
	Isoandrographolide	−8.2	−6.8	−8.0	−7.6
	12*S*-Hydroxyandrographolide	−7.9	−6.0	−7.5	−7.0
	Paniculide B	−7.1	−6.9	−6.9	−6.9
	Andrographolactone	−8.8	−5.9	−7.4	−7.3
	3-*O*-*β*-D-Glucopyranosyl andrographolide	−9.0	−7.2	−8.0	−8.0
Flavonoids	Andrographidin A	−8.6	−7.6	−7.7	−8.0
	Andrographidine E	−8.3	−6.7	−7.3	−7.9
	Andrographidine C	−8.6	−7.6	−8.0	−8.1
	5, 4’-Dihydroxy-7, 8-dimethoxyflavone	−7.5	−7.5	−7.6	−7.7
	Dihydroscullcapflavone I	−7.6	−7.0	−7.2	−7.1
	5-Hydroxy-7, 8-dimethoxy flavanone	−8.0	−7.2	−7.4	−7.3
	Apigenin-7, 4’-dimethylether	−7.9	−7.8	−7.8	−7.6
	5-Hydroxy-7, 2’, 6’-trimethoxy flavone	−7.7	−6.5	−7.0	−7.1
	1-2-Dihydroxy-6-8-dimethoxy xanthone	−7.5	−7.0	−7.1	−6.8
	Methylswertianin	−7.9	−6.6	−7.1	−6.8
	5-2’-6’-Trihydroxy-7-methoxyflavone	−8.2	−6.9	−7.5	−7.9
	5-2’-Dihydroxy-7-8-dimethoxyflavone	−8.2	−7.0	−7.7	−8.2
	5-7-4’-Trihydroxyflavone	−8.4	−7.8	−7.9	−7.2
	Isoswertisin	−8.4	−7.4	−7.7	−7.4
	Luteolin	−8.5	−8.3	−8.4	−8.0

**Table 3:** KEGG pathways of diterpene lactones and flavonoids.

Pathway Name	KO ID	Input Number	Leaf vs. Stem DEGs	Leaf vs. Root DEGs	Stem vs. Root DEGs
Phenylalanine metabolism	Ko00360	55	7	16	11
Terpenoid backbone biosynthesis	Ko00900	93	5	20	20
Diterpene biosynthesis	Ko00904	23	5	11	11
Phenylpropanoid biosynthesis	Ko00940	114	17	48	37
Flavonoid biosynthesis	Ko00941	26	2	10	8
Flavone and flavonol biosynthesis	Ko00944	3	-	-	-

## Data Availability

The datasets presented in this study can be found in online repositories. The names of the repository/repositories and accession number(s) can be found below: https://www.ncbi.nlm.nih.gov/ (accessed on 5 May 2023), PRJNA976906.

## Supplementary Materials

The following supporting information can be downloaded at https://www.mdpi.com/article/10.3390/molecules29040852/s1.
