# Supplementary material for: Integrating Transcriptome and Chemical Analyses to Provide Insights into Biosynthesis of Terpenoids and Flavonoids in the Medicinal Industrial Crop Andrographis paniculate and Its Antiviral Medicinal Parts
Source: Molecules. 2024 Feb 14;29(4):852. doi: 10.3390/molecules29040852 (PMC10893308; doi:10.3390/molecules29040852)
Supplement: Supplementary file 1 [file molecules-29-00852-s001.zip › Supplementary Figures.pdf]

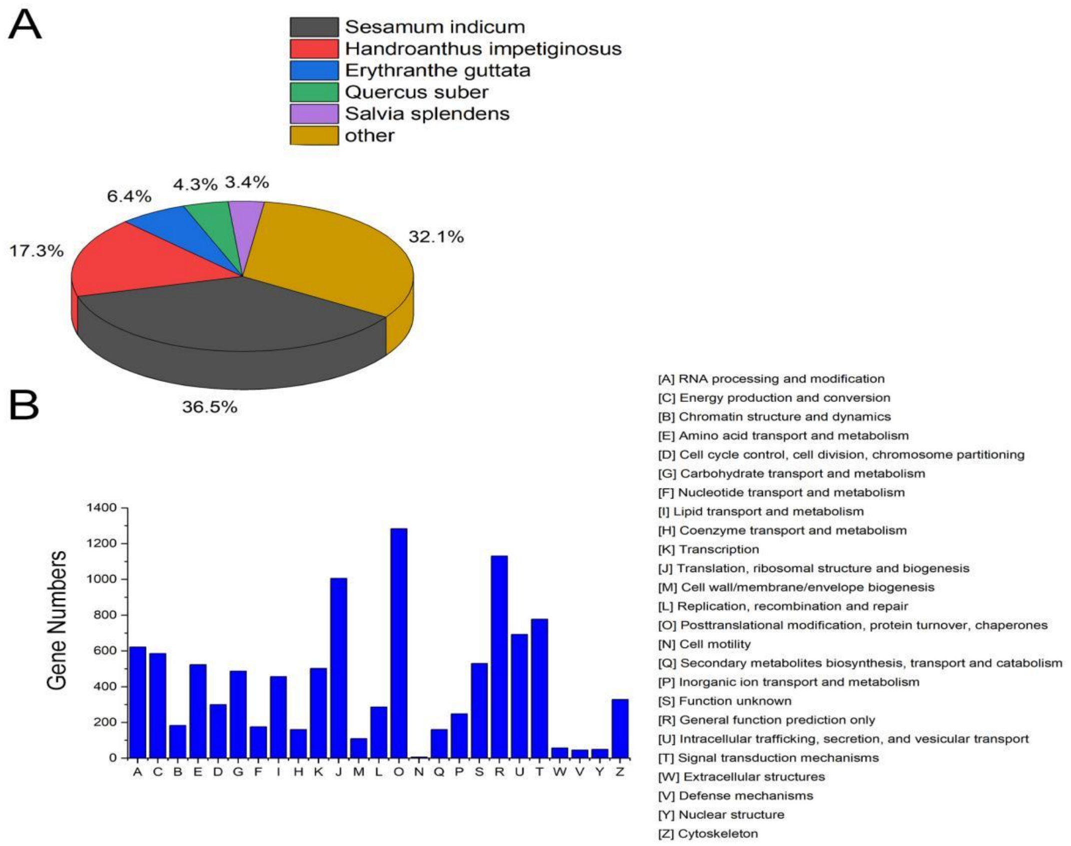

Supplementary Figure S1. Database annotation classification. (**A**) Species distribution of transcriptomic unigenes against NR database. (**B**) KOG classification.

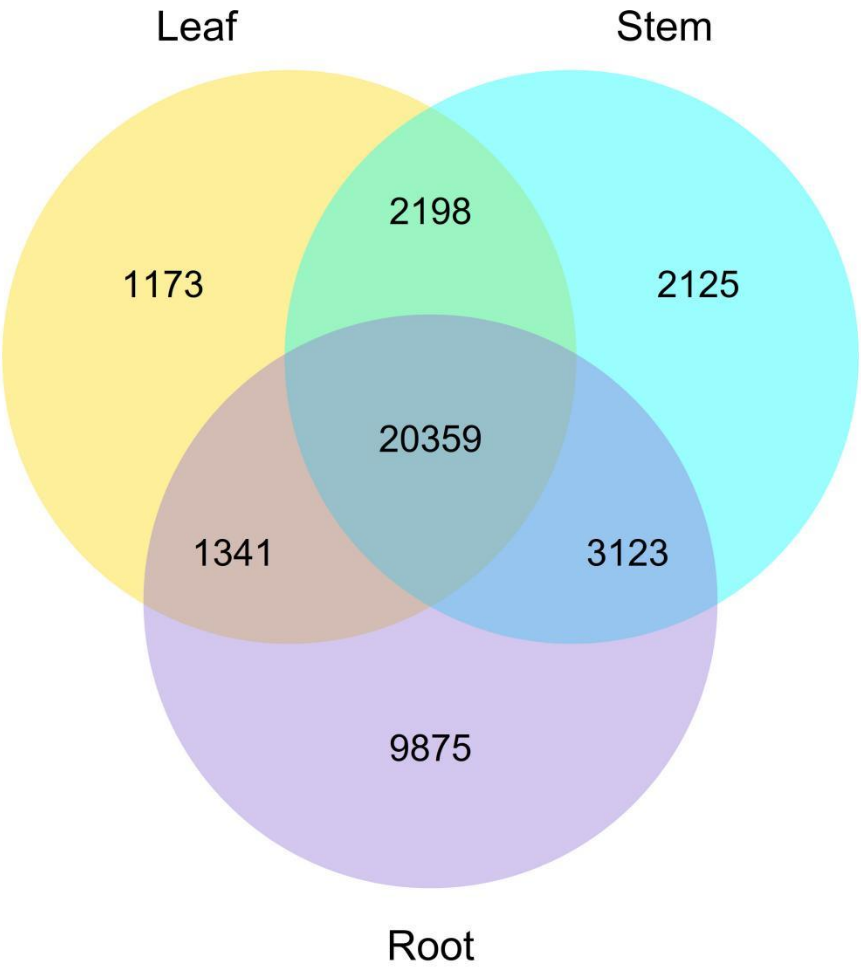

Supplementary Figure S2. Venn diagram of coexpressive unigenes in leaves, stems and roots of *A. paniculata*.

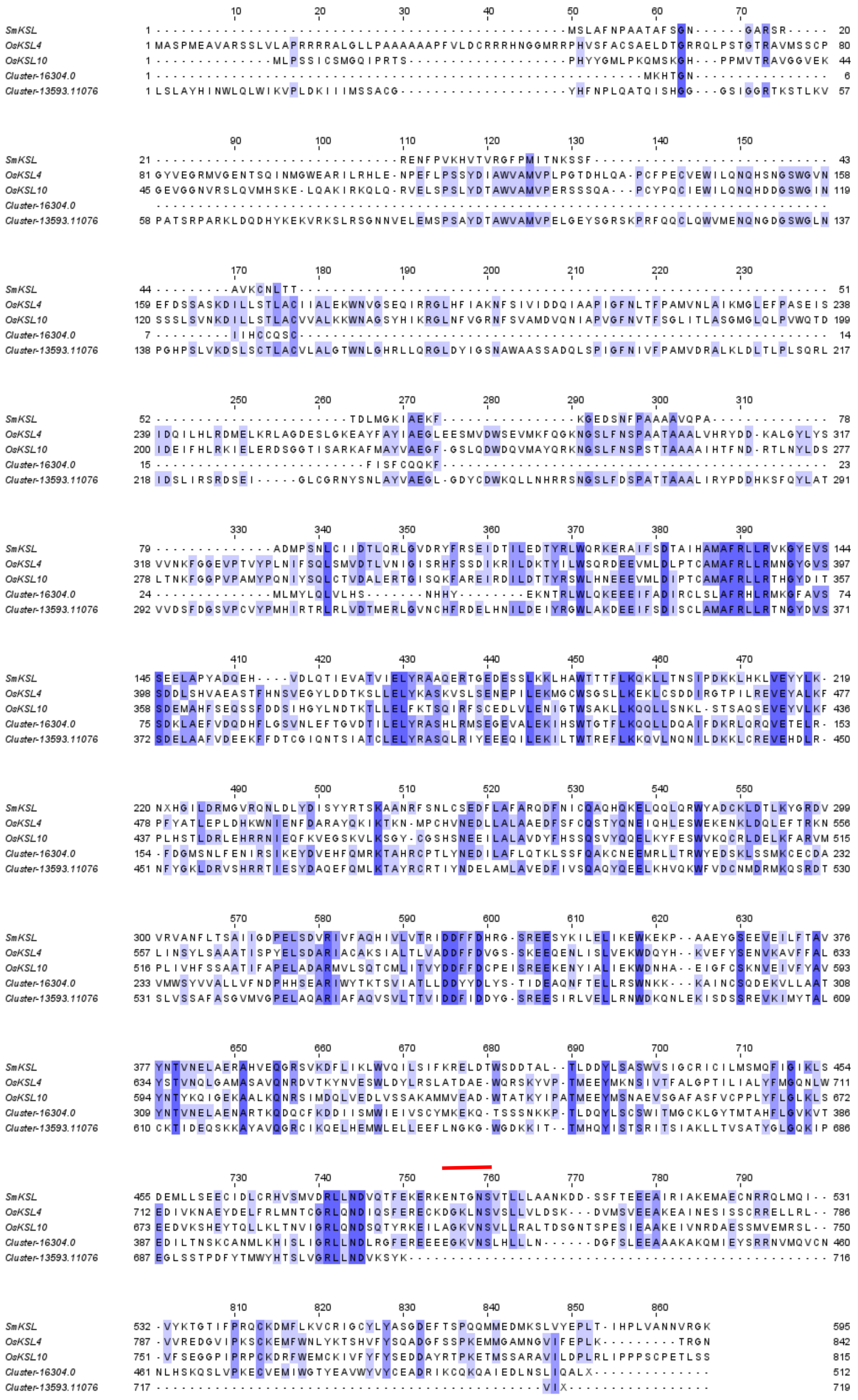

Supplementary Figure S3. Two sequence comparison of the *A. paniculata* kaurene synthase-like (KSL-like) enzymatic family with three previously characterized KSL enzymes from *Oryza sativa* and *salvia miltiorrhiza*. Underline indicates the characteristic DDxxD motif.
